# Mesoscale Characterization of Fracture Properties of Steel Fiber-Reinforced Concrete Using a Lattice–Particle Model

**DOI:** 10.3390/ma10020207

**Published:** 2017-02-21

**Authors:** Francisco Montero-Chacón, Héctor Cifuentes, Fernando Medina

**Affiliations:** 1 Department of Engineering, Universidad Loyola Andalucía, Calle Energía Solar 1, 41014 Sevilla, Spain; 2 ETS de Ingeniería, Universidad de Sevilla, Camino de los Descubrimientos s/n, 41092 Sevilla, Spain; bulte@us.es (H.C.); medinaencina@us.es (F.M.)

**Keywords:** lattice–particle model, fiber-reinforced concrete, fracture

## Abstract

This work presents a lattice–particle model for the analysis of steel fiber-reinforced concrete (SFRC). In this approach, fibers are explicitly modeled and connected to the concrete matrix lattice via interface elements. The interface behavior was calibrated by means of pullout tests and a range for the bond properties is proposed. The model was validated with analytical and experimental results under uniaxial tension and compression, demonstrating the ability of the model to correctly describe the effect of fiber volume fraction and distribution on fracture properties of SFRC. The lattice–particle model was integrated into a hierarchical homogenization-based scheme in which macroscopic material parameters are obtained from mesoscale simulations. Moreover, a representative volume element (RVE) analysis was carried out and the results shows that such an RVE does exist in the post-peak regime and until localization takes place. Finally, the multiscale upscaling strategy was successfully validated with three-point bending tests.

## 1. Introduction

Concrete is today’s most used construction material [1], and it has been used in many different applications for its versatility and techno-economic advantages. For instance, it allows the construction of structures of any shape and high compressive strength at a fraction of the cost of other important construction materials. On the other hand, its main drawback is its low tensile strength; however, this can be overcome with the inclusion of additional phases such as steel rebars [2], natural or artificial fibers [3], or carbon nanotubes [4], to cite a few.

The demand for high performance concrete (HPC) in civil infrastructures has thus increased and, moreover, empowered the development of ultra-high performance concrete (UHPC) [5] aiming at extraordinary material features, especially in terms of strength and durability.

The inclusion of fibers inside the quasi-brittle concrete matrix enhances the mechanical properties (e.g., strength, toughness, and fatigue life) of the new resulting composite material. In general, the mechanical response of the FRC depends on fiber parameters (size, stiffness, strength, volume content and shape), matrix parameters (stiffness, strength and fracture energy), and fiber–matrix bond parameters (bond strength, stiffness and debonding energy) [3]. Therefore, it is desirable that the FRC models account for these material parameters.

As the use of fiber-reinforced concrete (FRC) becomes more extended, the need for reliable tools to better understand its behavior increases. It is within this context that numerical models play an important role. Certainly, these are less time-demanding (and thus less expensive) than the experimental campaigns required to characterize FRC mixes. On the other hand, the physical phenomena governing the behavior of FRC must be completely understood so as to solely rely on computational models as a mean for developing new materials in silico, as proposed by the Integrated Computational Materials Engineering (ICME) philosophy [6]. In any case, experimental tests are an integral part of computational materials science.

Certainly, numerical models for the simulation of FRC have experienced important advances. Regarding continuum-based models, different approaches have been followed, such as smeared-crack models with homogenized FRC material properties [7,8] or embedded fibers [9]; cohesive models [10,11] which can be implemented within an extended-finite element method framework (XFEM); partition of unity finite element method (PUFEM) [12] which allows for the definition of fiber, matrix, and bond explicit constitutive behaviors; or multiscale approaches such as the micromorphic model [13] that takes into account the mesostructural level associated to the fiber–matrix interface process at the structural level, or homogenized mesolevel constitutive laws in damage models [14], to cite a few.

Discrete models, on the other hand, have shown their suitability for the analysis of fracture mechanics of concrete and other quasi-brittle materials, see for instance refs. [15,16,17,18,19,20,21]. For this reason, it has become an appropriate framework for the extension to FRC models, provided fibers can be treated as discrete entities via rigid-body-spring networks [22,23,24], pure lattice models [25,26] or lattice–particle models [27,28].

In this work, the lattice–particle model developed by the authors [28,29] is enhanced for the analysis of FRC. One main feature of this model is that fibers are explicitly modeled, i.e., the fibers have their own degrees of freedom rather than lumping their arresting effect on the element boundary, as in [22,27]. This is achieved by means of special fiber–matrix interface elements that, in contrast, must be characterized by pullout tests. Moreover, the use of a lattice–particle approach allows for the reduction of the number of degrees of freedom by accounting for different material responses. The model also considers the mechanical and geometrical properties of the constituents of FRC (e.g., mix properties, fiber size and distribution, and material properties) and it has been validated with analytical and experimental results. In this sense, not only the tensile and flexural behaviors have been analyzed, but also the compressive behavior, which has been less studied with numerical models.

Finally, the presented model provides a framework for its integration in multiscale analysis of FRC structures. A main concept in multiscale analysis is the existence and definition of a representative volume element (RVE), which has been discussed for plain concrete [29,30,31]. The effect of fiber reinforcement on the determination of an RVE, especially in the softening regime, is analyzed herein by means of an extensive numerical campaign.

## 2. Lattice–Particle Model for Fiber-Reinforced Concrete

In this section, the main features of the lattice–particle model for the fracture analysis of FRC at the mesoscale (i.e., ~10 mm) are presented. The model is based on previous concepts in [17,18,19,20,21], and it was firstly implemented for plain concrete in [29].

### 2.1. Mesostructure Generation

At the mesoscale, FRC presents a heterogeneous material structure that includes different phases: mortar, coarse aggregates, interfacial transition zone (ITZ) between these two, and fibers. Therefore, these have to be taken into account in the material generation.

In the first place, a coarse aggregate (i.e., maximum aggregate size, *d_max_* > 4.75 mm) distribution is generated following the procedure described in [20], which considers the mix properties (e.g., water to cement (*w*/*c*) ratio or aggregates content). A Fuller’s parabola with exponent *n* = 0.5 is used for the sieve curve [32] and, as proposed in [33], a 40% volume content of aggregates is assumed. The generated particles are placed within the domain using the take-and-place method [19,20] and, for the sake of simplicity, spherical shapes are considered. During this process, particle overlaps are not allowed.

The main input for the fiber generation is the volume content (*V_f_*), which, in addition to the fiber length (*L_f_*) and diameter (*d_f_*), provides the initial number of one-dimensional inclusions to be placed within the concrete matrix. Fiber elements are introduced at an initial point, within the specimen’s domain, and they are assigned a direction. Both variables are set following a pseudo-random uniform distribution. If the end of the fiber lies out of the boundaries, it is automatically trimmed and the subtracted volume is accounted for when placing the next fiber, preserving the initial *V_f_*. Since the aspect ratio of these inclusions is large, no overlap checks are performed at this point.

**Figure 1:**
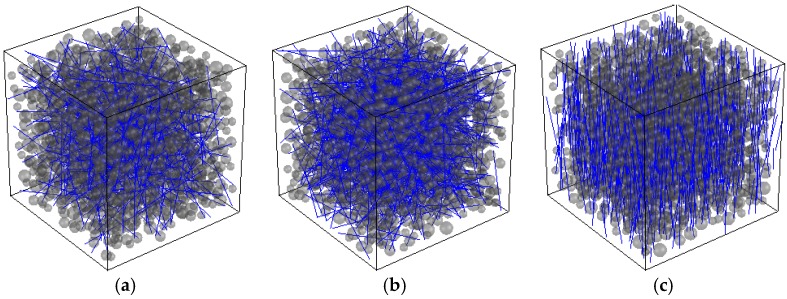
FRC numerical mesostrucures: (**a**) *V_f_* = 0.5% and *θ* = 90°; (**b**) *V_f_* = 1.0% and *θ* = 90°; and (**c**) *V_f_* = 1.0% and *θ* = 10°.

Fibers orientation is biased by the casting direction and rheology of the flow [34]. Thus, a misorientation angle (*θ*), which controls the maximum angle of the fiber with respect to a specific casting direction, is introduced in order to account for different orientation levels: perfectly oriented fiber distribution with respect to the casting direction (*θ* = 0°), partially oriented (0° < *θ* < 90°) or totally misoriented (*θ* = 90°). Figure 1 presents different numerically generated FRC specimens with different *V_f_* and *θ*.

### 2.2. Mesomechanical Elastic Behavior

The mechanical model of the current lattice–particle model lies on the interaction between particles and fibers through one-dimensional elements. For this reason, it is necessary to generate a mesh for the concrete matrix, fibers, and fiber–matrix interfaces.

**Figure 2:**
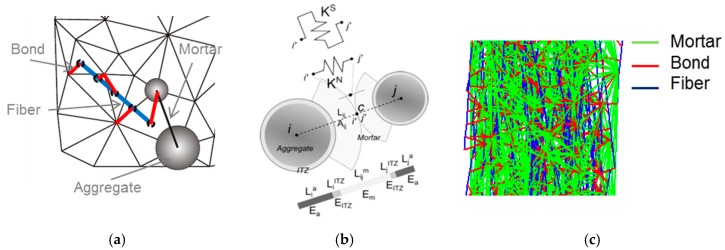
Mesomechanical features of the lattice–particle model: (**a**) concrete matrix Delaunay’s triangulation and fiber–matrix interaction; (**b**) aggregates interaction at the contact point and spring elements; and (**c**) resulting matrix, bond, and fiber mesh.

The concrete matrix mesh is constructed following a Delaunay’s triangulation using the centroids of the aggregates as in [20] (see Figure 2a). Therefore, the mesh size depends on the aggregates arrangement and every element represents the interaction between two aggregates and their corresponding influence zones, and the area is defined as:

(1) $$A_{ij}=\min\left(\pi r_{i}^{2},\pi r_{j}^{2}\right),$$

where *r_i_* and *r_j_* are the radii of aggregates *i* and *j*, respectively.

Along with the external nodes of the fibers, some internal nodes are generated in order to link the fibers to the matrix. The fiber element size is defined in terms of the matrix mesh size so as to maintain the average element size of the matrix. In the case of circular cross-section, the area of the fiber elements is defined as:

(2) $$A_{f}=\frac{\pi d_{f}^{2}}{4},$$

As mentioned above, the fiber nodes are linked to the closest aggregates through special interface elements, which represent the bond between the concrete matrix and the fibers. The bond elements area is defined as:

(3) $$A_{b}=\pi d_{f}L_{f},$$

Every element of the matrix mesh represents the mechanical interaction between the aggregates at the contact point, as depicted in Figure 2b. A set of spring elements acting the in normal and tangential direction, as in [18], is located at this contact point. The normal and tangential stiffness are:

(4) $$K_{ij}^{N}=\alpha_{1}\frac{E_{ij}A_{ij}}{L_{ij}},$$

(5) $$K_{ij}^{S}=\alpha_{2}\frac{E_{ij}A_{ij}}{L_{ij}},$$

where *α*_1_ and *α*_2_ are the normal and shear parameters used to adjust the macroscopic elastic modulus and Poisson’s ratio; and their typical values are 1 and 0.2, respectively. On the other hand, *E_ij_* is the local (i.e., element) elastic modulus and *L_ij_* is the length of the element directly obtained from the triangulation.

The local elastic modulus is defined in terms of the three phases that are considered in the concrete matrix, mortar (*E_m_*), coarse aggregates (*E_a_*), and ITZ (*E_ITZ_*), as well as on the aggregate radii, the ITZ thickness of each aggregate (*L_i_^ITZ^* and *L_j_^ITZ^*), and the mortar length (*L_ij_^m^*), which is the rest of the element. Assuming a serial coupling along the element [12], the local elastic modulus reads:

(6) $$\frac{L_{ij}}{E_{ij}}\;=\;\frac{r_{i}}{E_{a}}\;+\;\frac{L_{i}^{ITZ}}{E_{ITZ}}\;+\;\frac{L_{ij}^{m}}{E_{m}}\;+\;\frac{L_{j}^{ITZ}}{E_{ITZ}}\;+\;\frac{r_{j}}{E_{a}},$$

The elastic modulus for cement mortars varies from 10 GPa to 70 GPa, depending on the porosity [35]; and that of coarse aggregates ranges from 70 GPa to 90 GPa [35,36]. For the numerical simulations, the material properties considered are obtained from [20], although lower scale discrete models can be also used to compute these [14].

Fibers are modeled as truss elements, with the following axial stiffness:

(7) $$K_{f}^{N}\;=\;\frac{E_{f}A_{f}}{L_{f,e}},$$

where *E_f_* is the elastic modulus of the fiber, and *A_f_* the cross-sectional area of the fiber, given by Equation (2).

The bond elements (Figure 2c) are defined as pure shear elements, since no axial interaction is expected at the interface:

(8) $$K_{b}^{S}\;=\;\frac{E_{b}A_{b}}{L_{b}},$$

In general, the normal and tangential displacements of an element, **u***_n_* and **u***_s_*, respectively, can be computed as:

(9) $$u_{n}\;=\;\left(u\cdot n\right)n,$$

(10) $$u_{s}\;=\;u-u_{n},$$

where **u** is the element displacement vector and **n** is the normal vector that, for an element connecting nodes *i* and *j*, is defined as:

(11) $$n\;=\;\frac{x_{j}-x_{i}}{L_{ij}},$$

with **x***_i_* and **x***_j_* the coordinates of nodes *i* and *j*, respectively.

The normal and tangential element strains can be obtained as:

(12) $$\varepsilon_{n}\;=\;\frac{\left|u_{n}\right|}{L_{ij}},$$

(13) $$\varepsilon_{s}\;=\;\frac{\left|u_{s}\right|}{L_{ij}},$$

and, finally, the normal and shear stresses can respectively be computed from:

(14) $$\sigma \;=\;\alpha_{1}E\varepsilon_{n},$$

(15) $$\tau =\alpha_{2}E\varepsilon_{s},$$

### 2.3. Mesoscale Fracture Behavior

**Figure 3:**
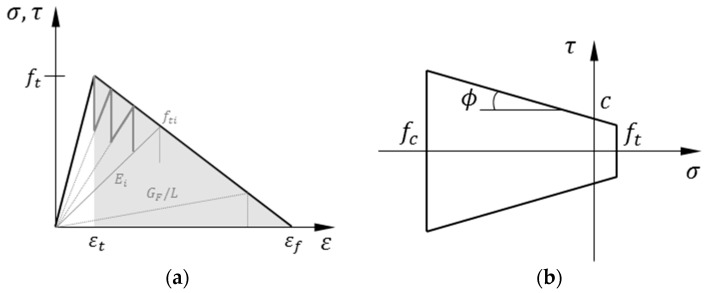
Mesoscale fracture behavior of matrix elements: (**a**) linear softening curve with sequential reduction; and (**b**) Mohr–Coulomb fracture surface with tension cut-off and compression cap.

In order to account for the material non-linearity intrinsic to fracture, a sequentially event-driven solution scheme [37], in which the non-linear problem is solved stepwise into several linear analyses, is used. Thus, at every simulation step, unitary prescribed forces (or displacements) are applied to the specimen and the element stress pairs are calculated. In the case of the concrete matrix, a linear softening curve is considered (although other shapes can be implemented). This law is discretized into *N* segments (see Figure 3a), and the elastic modulus of the element with the maximum stress-to-strength ratio is updated following this expression:

(16) $$E_{i}=\frac{f_{t,i}}{\varepsilon_{t}-\left(i-1\right)\left(\frac{\varepsilon_{f}-\varepsilon_{t}}{N}\right)},$$

where *i* is the current segment; *E_i_* and *f_t,i_* are the current elastic modulus and tensile strength, respectively; *ε_t_* is the initial cracking strain; and *ε_f_* is the ultimate tensile strain which is defined in terms of the element length and the fracture energy, *G_F_*, to avoid the mesh-dependency issues:

(17) $$\varepsilon_{f}=\frac{2G_{F}}{f_{t}L}+\frac{f_{t}}{E_{0}},$$

As observed in Figure 3a, with the discretization of the softening curve, some fracture energy is lost. Such energy loss can be minimized by increasing the number of segments, *N*. On the other hand, a corrective term ς is included such that an updated value for the fracture energy is used, $G\prime_{F}\;=\;\varsigma^{2}G_{F}$, which is equivalent to increasing the tensile strength and ultimate strain ς times, as proposed in [37]. As a reference, for a total number of segments *N* = 10, the corrective term yields ς~1.1.

In the case of bond and fiber elements, a general bilinear material behavior is implemented so as to account for a wide range of behaviors, namely from softening to hardening. This is achieved by defining initial and ultimate stress–strain pairs, and the sequential reduction takes place therein, as explained above.

Since shear interaction is present in matrix and bond elements, a Mohr–Coulomb failure surface with tension cut-off and compression cap (Figure 3b) is used, as proposed in [17]. This surface is defined by the tensile strength, *f_t_*; the slope of the failure envelope or angle of internal friction, *ϕ*; the cohesion stress, *c*; and the compressive strength, *f_c_*.

Although the ITZ is known to be the weakest link in the concrete mesostructure, it has little influence on the macroscopic tensile strength [38], in contrast to mortar properties. Therefore, local fracture parameters in the matrix elements are those for mortar. In this regard, tensile strength in common mortars vary from 2 MPa up to 15 MPa [36]. For granular materials, internal friction angles are in the range of 25° to 45° [39]. The cohesion stress is set as *c* = 2*f_t_*. It must be remarked that, although this mortar information is lumped into the spring sets, it could be explicitly modeled by including mortar particles, and thus decreasing the *d_max_* value of the generated distribution.

Even though fracture events are more likely to happen in matrix and bond elements, due to their lower strength, fibers are assigned a Rankine failure criterion characterized by the fiber yield strength.

### 2.4. Meso-Macro Upscaling Strategy

The fiber-reinforced lattice–particle model can be used to upscale the mesoscale properties to the macroscale (i.e., ~1 m), in a numerical homogenization fashion [40]. However, in the case of quasi-brittle materials, that show strain localization, this step is not straightforward and some considerations must be taken into account.

One main concept in homogenization-based schemes is the so-called representative volume element (RVE) that, basically, is a volume element over which a measured value becomes representative of the whole. In plain concrete, the definition of such an RVE is not possible due to strain localization [29,30], but can be overcome by the definition of failure average zones [31]. However, FRC may present in some cases a pseudo-plastic behavior, and thus circumventing this problem. It is within this context that numerical homogenization can be used as a multiscale approach so as to evaluate the macroscopic behavior of FRC structures.

In this work we propose the discrete to continuum upscaling procedure presented in [14], by which the material properties of the continuum damage plasticity (CDP) model are obtained by virtual testing. Thus, the tensile cracking strain, which is required by the CDP model to describe the tensile behavior of the material, can be obtained as:

(18) $${\tilde{\varepsilon }}_{t}^{ck}=\varepsilon_{t}-\frac{\sigma_{t}}{E_{0}},$$

where *ε_t_* is the total tensile strain, *σ_t_* is the tensile stress and *E*_0_ is the elastic modulus, obtained by means of mesoscale simulations.

## 3. Results and Discussion

The model presented in Section 2 considers the matrix, fiber, and fiber–matrix interaction parameters required to describe the mechanical behavior of FRC, as suggested in [3]. Therefore, it requires the input of 15 material properties and the material specifications (e.g., mix information, and fiber distribution). These properties are summarized in Table 1.

In this section, the fiber-reinforced lattice particle model is calibrated and validated by means of different tests. In the first place, pullout tests are carried out to characterize the fiber–matrix interface behavior, which is essential in the response of FRC models. Next, uniaxial tensile and compressive tests are carried out so as to validate the model. Finally, the upscaling approach described in Section 2.4 is validated via three-point bending tests (3PBT), showing the ability of the model to reproduce the fracture behavior FRC.

### 3.1. Pullout Test

**Figure 4:**
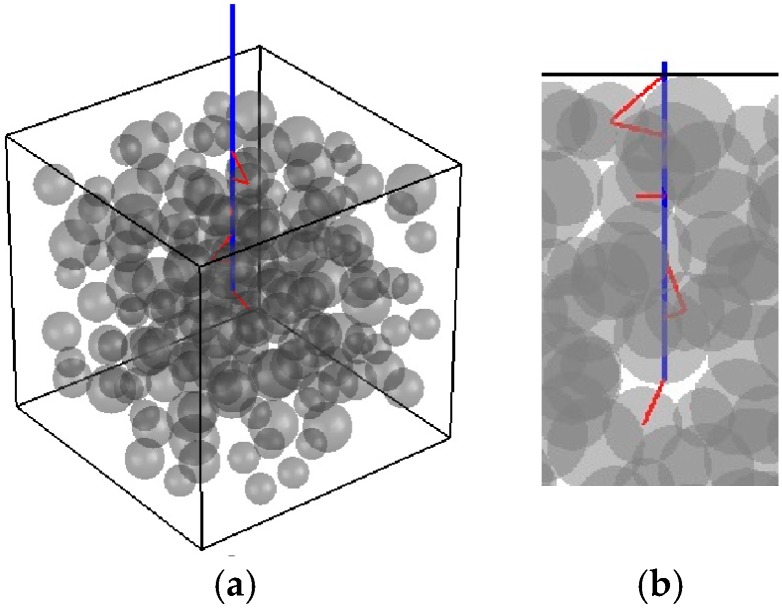
Fiber pullout test numerical simulation setup: (**a**) the fiber (in color blue) is partially embedded in the concrete matrix and it is pulled out by its free end; and (**b**) the pullout test allows for the characterization of the interface elements (in color red).

The fiber–matrix interface behavior is best characterized by means of fiber pullout tests [3]. In these tests, the fiber is partially embedded in the matrix and it is pulled out through its free end, as depicted in Figure 4. The global response is governed by the so-called force-slip curve, and three main stages can be observed in this curve: (1) an elastic branch prior to the chemical strength degradation; (2) non-linear fiber–matrix debonding; and (3) frictional softening [3].

The present model accounts for different force-slip behaviors: slip hardening, perfectly elastic-plastic, and slip softening. These are implemented via bilinear laws that require four material properties, as described in Table 1.

Several pullout tests were simulated and compared to the results by Cunha et al. [41] and Kim et al. [42]. In these steel fiber pullout simulations, different concrete specimens with different fiber sizes were considered, in accordance with [41,42]. The specimens were fixed at the bottom surface and the free end of the half-embedded steel fiber was pulled-out.

**Figure 5:**
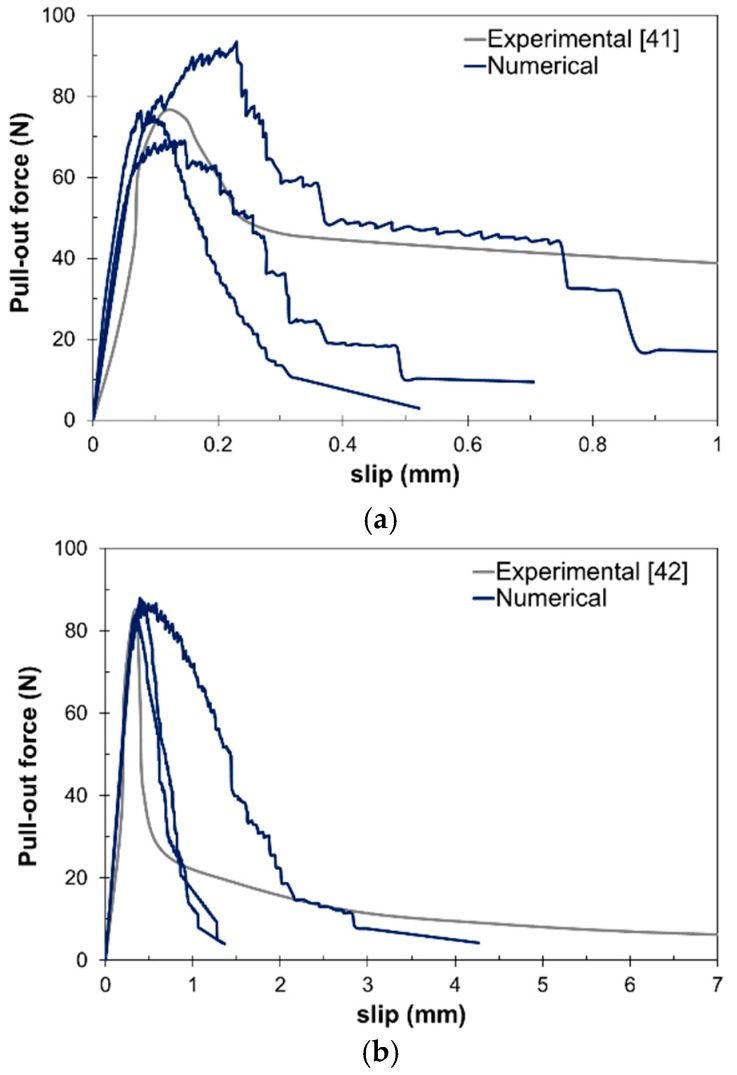
Fiber pullout test numerical simulation results: (**a**) low slip range; and (**b**) high slip range.

Figure 5 shows the results for the pullout simulations. As can be observed, the model is able to reproduce well the pullout forces at low slip values, i.e., below 1 mm (Figure 5a). On the other hand, the model is also able to develop large slip values (Figure 5b). Table 2 presents the optimal ranges for the interface material properties that are used in further simulations.

### 3.2. Tensile Tests

Uniaxial tensile tests provide important information regarding the fracture properties of FRC, for instance the tensile strength (*f_t_*) and fracture energy (*G_F_*). Moreover, the effect of fiber volume content and distribution can be observed.

In this work, the uniaxial tensile response of steel fiber-reinforced concrete (SFRC) is validated using the analytical model proposed by Karihaloo and Lange-Kornbak [43] and experimental double-edge-notched (DEN) tension tests by Gopalaratnam and Shah [44]. On the other hand, the influence of *V_f_* and *θ* in the tensile response is analyzed by means of unnotched uniaxial tension tests.

#### 3.2.1. Elastic Modulus

In the first place, and before characterizing the fracture behavior of SFRC, the elastic modulus of the composite material was determined following an automatic numerical campaign, evaluating a total of 100 prismatic specimens with size 50 × 25 × 150 mm^3^, *d_max_* = 8 mm, steel fibers with *L_f_* = 35 mm and *d_f_* = 0.5 mm, random distribution and variable *V_f_* = 0.0%–3.0%. The elastic properties of the phases were *E_m_* = 20 GPa, *E_a_* = 70 GPa, *E_f_* = 210 GPa, and *E_b_* = 5 GPa.

**Figure 6:**
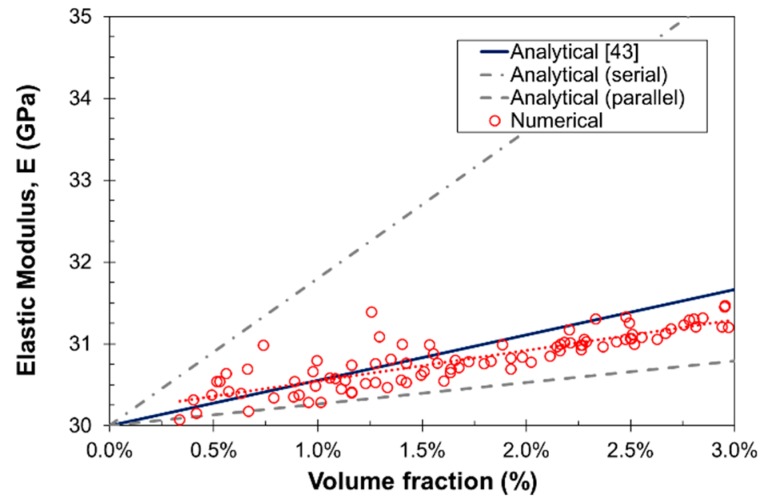
Effect of *V_f_* in the elastic modulus of SFRC.

In Figure 6, the numerical results are compared to the analytical expression from [43] and the rule of mixture bounds for composite materials. It can be observed that the numerical results are in agreement with those obtained from [43] and the trend is similar to that of the parallel behavior, as expected. On the other hand, it can be also observed that as *V_f_* increases, the scatter decreases as a result of an increase in the number of nodes in the system.

#### 3.2.2. Tensile Strength

A total number of 50 specimens with the same dimensions and properties used for the elastic modulus analysis, were subjected to complete uniaxial tension tests. The fracture properties for the concrete matrix were *f_t_* = 4 MPa, *f_c_* = −12*f_t_*, *c* = 2*f_t_*, *ϕ* = 35°, and *G_F_* = 10 N/m; and a softening behavior for the bond determined by *τ*_1_ = 1 MPa, *τ*_2_ = 0.1 MPa, and *ε_f_*/*ε_i_* = 15 was considered. The volume fraction range was set to *V_f_* = 0.5%–1.5% so as to analyze the experimental configurations in more details.

**Figure 7:**
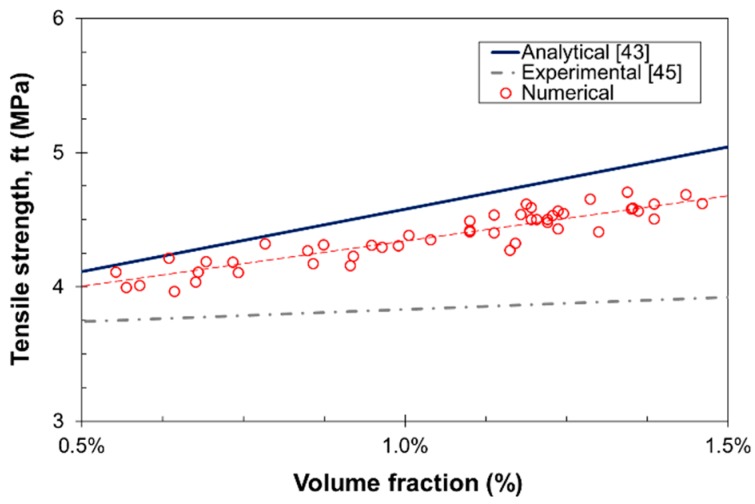
Effect of *V_f_* in the tensile strength of SFRC.

Figure 7 shows the numerical results for the tensile strength for different volume fractions. The results are in agreement with those obtained from the expression in [43]. The effect of *V_f_* in *f_t_* is slightly lower in the numerical simulations, but larger than those reported in [45].

Splitting tensile tests were carried out on cylindrical specimens of 100 mm in the laboratory, using the same concrete mix and fiber geometry as the simulations, and a value of *f_st_* = 4.97 MPa was obtained for *V_f_* = 0.6%. The equivalent tensile strength, obtained with the Model Code formula [46], is thus *f_t_* = 4.47 MPa, which is in agreement with the numerical result, *f_t_* = 4.21 MPa.

**Figure 8:**
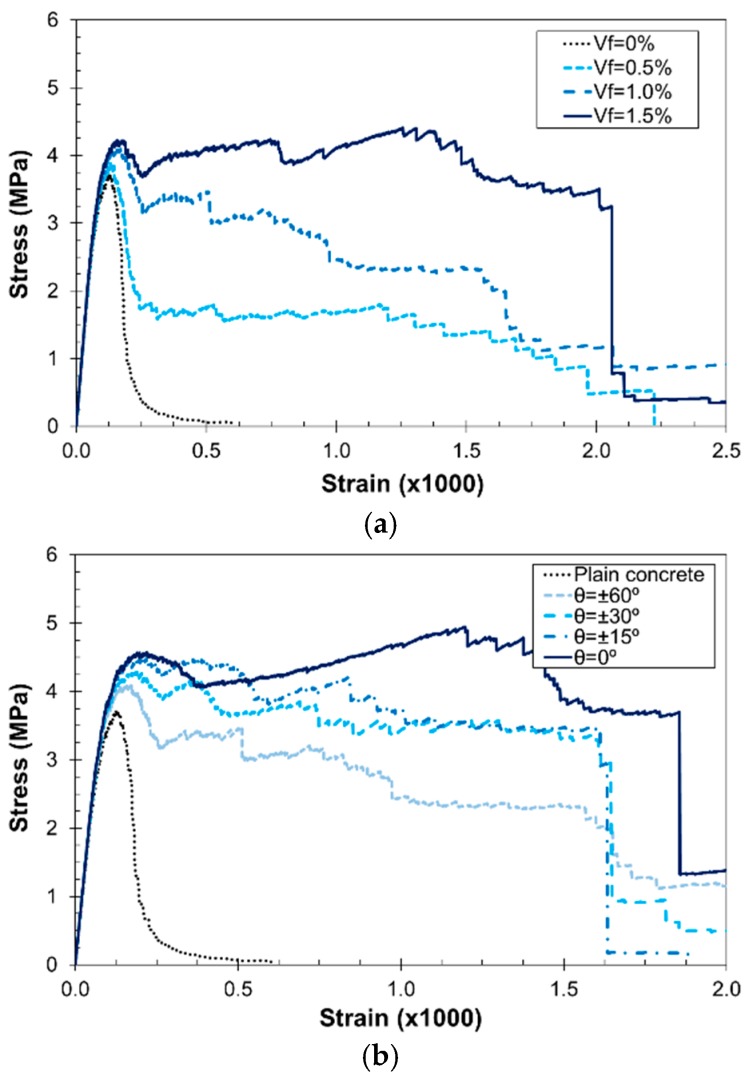
SFRC uniaxial tension stress–strain curves: (**a**) effect of *V_f_* with *θ* = 60°; and (**b**) effect of *θ* with *V_f_* = 1.0%.

The previous simulations serve as the basis for the analysis of the effect of *V_f_* and fiber orientation in the stress–strain uniaxial tension response. Figure 8 presents the effect on the tensile response of *V_f_* in random distribution (*θ* = 60°) and fiber orientation (for a given *V_f_* = 1.0%). In the first case (Figure 8a), as *V_f_* increases, the tensile strength and ductility increases. In the second case (Figure 8b), as the fibers become more aligned with respect to the loading axis, the strength and ductility increases. It must be remarked that this effect is more evident for high *θ* values; however, the tortuosity of the crack may diminish this effect for highly oriented distributions. In Figure 8b, it can be observed that for misorientations below 15°, the maximum arresting effect of the fibers is approached.

**Figure 9:**
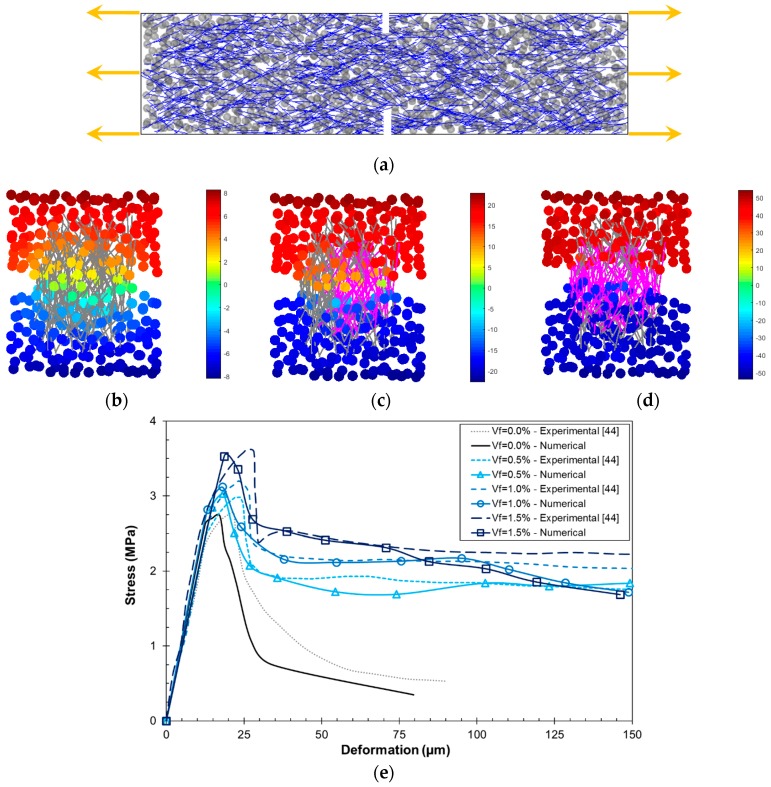
DEN tensile test simulations: (**a**) numerical mesostructure generation with 5 mm aggregates and steel fibers with *V_f_* = 1.0% and *θ* = 45°; (**b**) displacement field (in μm) at the pre-peak regime (15 μm deformation); (**c**) displacement field (in μm) at the post-peak regime (softening, 50 μm deformation) and acting fibers (in magenta); (**d**) displacement field (in μm) at the post-peak regime (residual capacity, 100 μm deformation) and acting fibers (in magenta); and (**e**) uniaxial tension stress–deformation curves for different *V_f_*.

A second type of uniaxial tension tests was simulated in order to validate the FRC lattice–particle model. Numerical DEN tensile tests, as in [44], were carried out and the resulting stress–deformation curves are compared to the experimental ones. The specimen size was 76 mm × 19 mm × 305 mm, with two notches of 13 mm × 3 mm, as depicted in Figure 9a. In these tests, the material information is different to the previous one. The concrete matrix has a *d_max_* = 5 mm, *f_t_* = 2.4 MPa, *f_c_* = −10*f_t_*, *c* = 2*f_t_*, *ϕ* = 35°, and *G_F_* = 10 N/m. Smooth steel fibers with *L_f_* = 25 mm and *d_f_* = 0.4 mm were used and the bond properties were *E_b_* = 5 GPa, *τ*_1_ = 2.5 MPa, *τ*_2_ = 0.1 MPa, and *ε_f_*/*ε_i_* = 15.

Figure 9b–d shows the longitudinal displacements at the notched are of the specimen for different levels of deformation, namely pre-peak at 15 μm (Figure 9b), post-peak at 50 μm (Figure 9c), and post-peak at 100 μm (Figure 9d). The acting fibers are highlighted in order to observe their effect throughout the crack formation process. In the pre-peak zone, microcracks occur within the matrix close to the notched zone, which is evidenced as a smooth displacement gradient. As the deformation increases, a macrocrack starts to develop which is arrested by the fibers located in that zone (Figure 9c). In this sense, a displacement jump is observed close to the notch. Finally, a wide crack is fully developed at high deformation values, and the fiber bridging zone covers the whole specimen’s section (Figure 9d).

The stress–deformation results are presented in Figure 9e. It can be observed that the model predicts well the tensile strength and its dependency with respect to *V_f_*, and the post-peak behavior for intermediate deformation values. It must be remarked that the use of linear softening curves at the mesomechanical level may restrict the shape of the stress–deformation curve, as observed for the plain concrete (*V_f_* = 0.0%) case. This can be tackled by implementing polylinear softening curves such as those proposed in [10], in order to adjust the tail of the curve. However, the results obtained by the linear model are acceptable and the simplicity is preserved.

#### 3.2.3. Fracture Energy and Characteristic Length

The uniaxial tension tests of the unnotched specimens were used to compute the fracture energy and, subsequently, determine the characteristic length (*l_ch_*) of these with:

(19) $$G_{F}=\int_{0}^{w_{max}}\sigma \left(w\right)dw$$

(20) $$l_{ch}=\frac{EG_{F}}{f_{t}^{2}}$$

where *w* is the crack width, *w_max_* the maximum crack width; determined from the deformation response after the peak.

**Figure 10:**
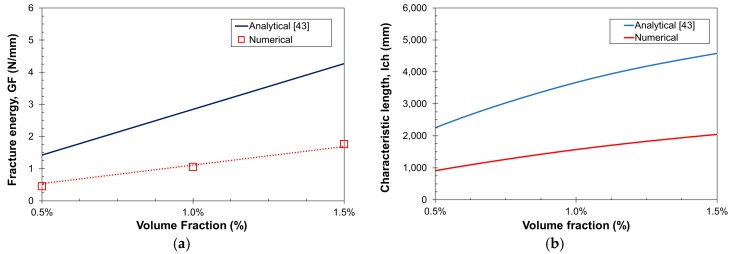
Effect of *V_f_* in (**a**) the fracture energy; and (**b**) the characteristic length of SFRC.

The computed *G_F_* and *l_ch_* are compared to the analytical results proposed in [43] in Figure 10. The numerical predictions for *G_F_* are lower than the analytical values (Figure 10a). This can be explained by the type of softening curve used at the mortar level, which can be enhanced so as to catch better the tail of the curves, and the fact that the integration of ductile curves presented by FRC, in which the softening tail is not always fully developed, is way more complex than that for plain concrete. Finally, the numerical characteristic length was derived from regression curves for *E*, *G_F_* and *f_t_*, and using Equation (20). Since *l_ch_* is directly proportional to *G_F_*, the differences in the latter are also brought into the former (Figure 10b).

### 3.3. Compression Tests

**Figure 11:**
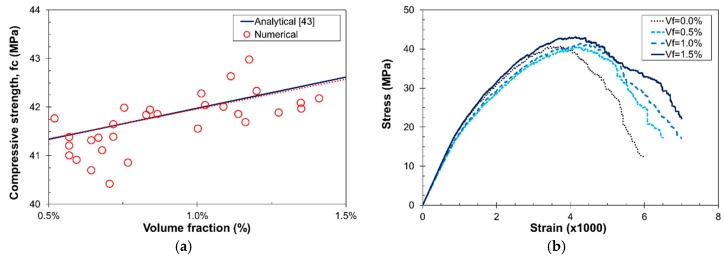
Effect of *V_f_* in the compressive strength of SFRC: (**a**) numerical predictions versus analytical model [33]; and (**b**) stress–strain curves for different *V_f_* and *θ* = 45°.

The uniaxial compression characterization is numerically more demanding than in tension, as the strain values are larger. For this reason, the same approach as in Section 3.2.2 was followed but for a total number of 30 simulations in cubic specimens with size 50 mm. The compressive strength dependence with respect to *V_f_* is presented in Figure 11. Figure 11a presents the numerical predictions for the compressive strength, which are in perfect agreement with those from the analytical expression by Karihaloo and Lange-Kornbak [43]. On the other hand, Figure 11b shows the stress–strain curves for different *V_f_* and, as shown in [3], the ductility of the composite material increases with this value.

**Figure 12:**
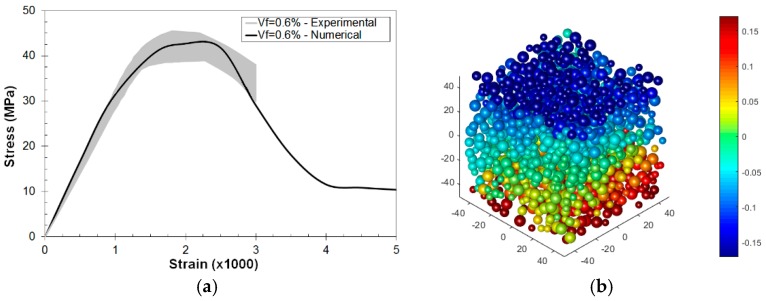
Uniaxial compression test: (**a**) experimental validation for *V_f_* = 0.6% and *θ* = 45°; and (**b**) vertical displacement field (in mm) at a total strain of 3% (post-peak).

Experimental tests were carried out in order to validate the compressive behavior. Cubic specimens with size 100 mm, same mix content and fiber geometry as in Section 3.2, and *V_f_* = 0.6% were considered. The static elastic modulus of concrete (*E_c_*) was determined according to UNE EN12390-13:2014, by gradually loading a cylindrical specimen in compression to approximately a third of its failure load and measuring the corresponding strain from 30 mm strain gauges. The experimental value, *E_c_* = 31.7 GPa (±7%), agrees well with the numerical prediction in Section 3.2.1, *E_c_* = 30.4 GPa, for the same mix. The compressive strength (*f_c_*) was determined following the UNE EN12930-3:2009 procedure, and the experimental value, *f_c_* = 41 MPa (±2%), is in perfect agreement with the numerical predictions (*f_c_* = 41.01 MPa). Figure 12a presents the uniaxial compression test stress–strain curve and, as can be observed, the numerical results are in agreement with the experimental data. Figure 12b presents the vertical displacement at a strain of 3%.

### 3.4. Macroscale Three-Point Bending Tests

The fiber-reinforced lattice–particle model presented in this work can be used at the mesoscale to characterize the material behavior, which can be used as input property at macroscale models in larger structures. In this step, the definition of the RVE is fundamental in order to ensure the representativity of the macroscopic results. Thus, in the first place, an analysis on the RVE size of FRC is carried out. Then, the meso- to macroscale upscaling approach presented in Section 2.4 is validated by means of three-point bending tests (3PBT).

#### 3.4.1. RVE Size Analysis

The determination of the RVE size for plain concrete for the elastic regime has been successfully analyzed in the literature [29,30,31]; and for the inelastic regime a redefinition of the RVE as a localization band has been presented [31]. In this work, the procedure followed in [29] for plain concrete has been extended to the case of FRC, reaching the inelastic regime. 

The effect of *V_f_* and *θ* on the determination of the RVE size is analyzed. For this purpose five specimen sizes (*D* = 35, 40, 50, 60, and 75 mm) with four different fiber volume contents (*V_f_* = 0.0%, 0.5%, 1.0%, and 2.0%) and four fiber orientations (*θ* = 0°, 30°, 60°, and 90°) were considered. Five realizations were made for each configuration, resulting in a total of 325 simulations. Uniaxial tensile tests with uniform boundary conditions were carried out and the measured quantities were the peak stress (i.e., tensile strength), *f_t_*_0_; and post-peak stress at 1% strain, *f_t_*_1_. Chi-square tests with 95% accuracy and statistical degree of freedom of 2 (χ^2^_95%_ = 0.103) were carried out.

**Figure 13:**
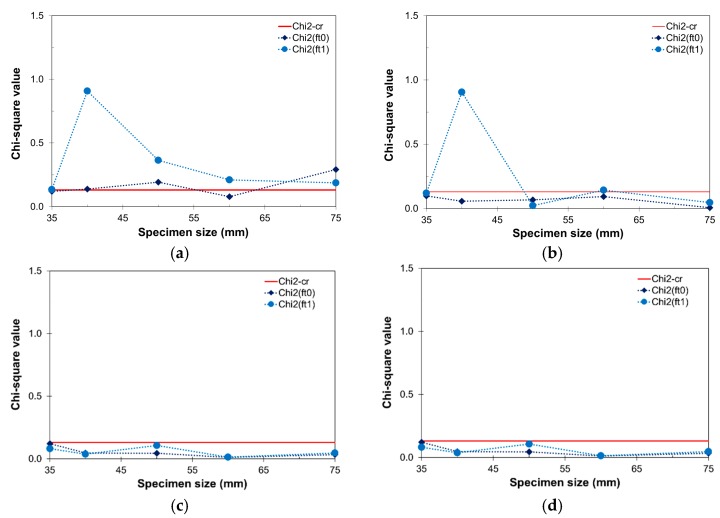
Chi-square tests for *f_t_*_0_ and *f_t_*_1_ and perfectly oriented fiber distributions (*θ* = 0°): (**a**) *V_f_* = 0.0%; (**b**) *V_f_* = 0.5%; (**c**) *V_f_* = 1.0%; and (**d**) *V_f_* = 2.0%.

In Figure 13 the Chi-square values for *f_t_*_0_ and *f_t_*_1_ are presented for different *V_f_* and perfectly oriented fibers. It is observed that, as *V_f_* increases, both quantities pass the test. The inelastic-related quantity, *f_t_*_1_, requires larger specimen sizes to stabilize. From the results, values of *D* > 60 mm and *V_f_* > 0.5%, and completely aligned fibers (*θ* = 0°) satisfy the RVE criteria. However, it must be remarked that this is valid only along the loading axis, while the transversal plane would lack fibers arresting the cracks and therefore would behave like plain concrete instead in those directions.

**Figure 14:**
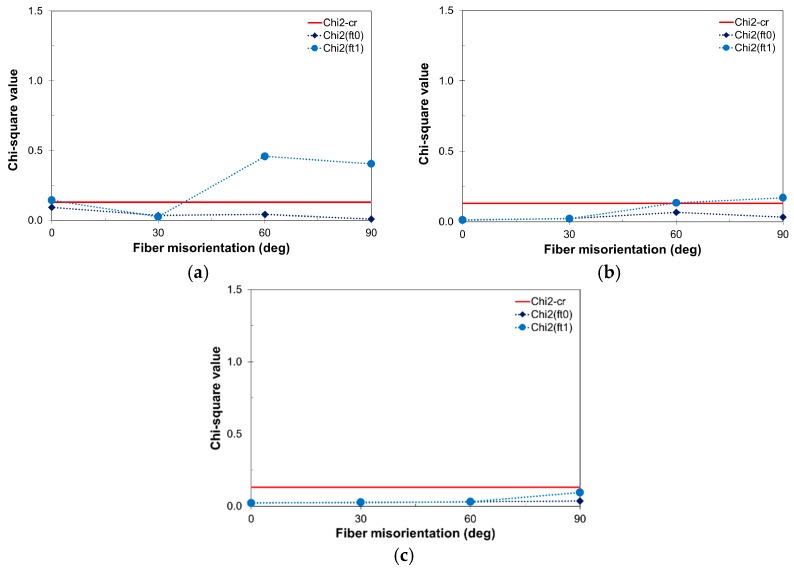
Chi-square tests for *f_t_*_0_ and *f_t_*_1_ on specimens of *D* = 60 mm and different *θ*: (**a**) *V_f_* = 0.5%; (**b**) *V_f_* = 1.0%; and (**c**) *V_f_* = 2.0%.

Regarding the fiber orientation, as observed in Figure 14, higher misorientation values result in larger RVE sizes. The peak quantity, *f_t_*_0_, is much less affected. Thus, for a specimen size of *D* = 60 mm, an RVE is found for any *θ* value when *V_f_* > 2.0%. However, in the range of *θ* = 30°–40°, which is the typical fiber misorientation observed in experiments [47], an RVE can be found for *V_f_* > 0.5%. On the other hand, *θ* has a strong influence on χ^2^(*f_t_*_1_), and limited influence on χ^2^(*f_t_*_0_) in contrast. This effect decreases as *V_f_* increases.

#### 3.4.2. Experimental Validation

Three-point bending tests (3PBT) were carried out on prismatic beams with a cross section of 100 mm × 100 mm, 400 mm of span, and a total length of 440 mm. The FRC composition was the same as in Section 3.3. The beams were notched with a thin (3 mm) diamond saw to a notch to depth ratio a/W = 1/6, according to EN 14651. The crack mouth opening displacement (CMOD) was measured with a clip gauge transducer and used as the feedback control signal. The load-point deflection was measured simultaneously by means of a linearly variable displacement transducer (LVDT) mounted on a rigid frame in order to avoid parasitic torsional effects on the measurement of vertical displacements. The tests were performed in a stiff closed-loop universal testing machine with a maximum load capacity of 50 kN.

**Figure 15:**
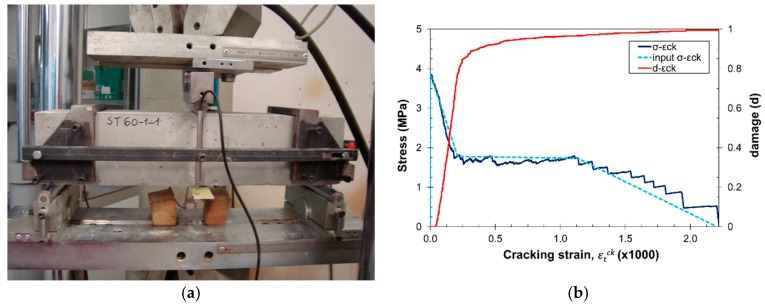
(**a**) 3PBT experimental setup; and (**b**) uniaxial tensile stress–cracking strain and tensile damage–cracking strain curve obtained for *V_f_* = 0.6% and *θ* = 30°.

The 3PBT numerical simulations were carried out in Abaqus using the concrete damage plasticity model [48], which has been successfully proven in these kind of analyses [14]. Figure 15a shows the experimental setup for the 3PBT. In 3PBT configurations, tensile behavior governs the global response of the structure, therefore the uniaxial tensile behavior of the material was obtained from lattice–particle simulations on specimens fulfilling the previous RVE size considerations, and the resulting stress–cracking strain and tensile damage–cracking strain curve were extracted (Figure 15b).

**Figure 16:**
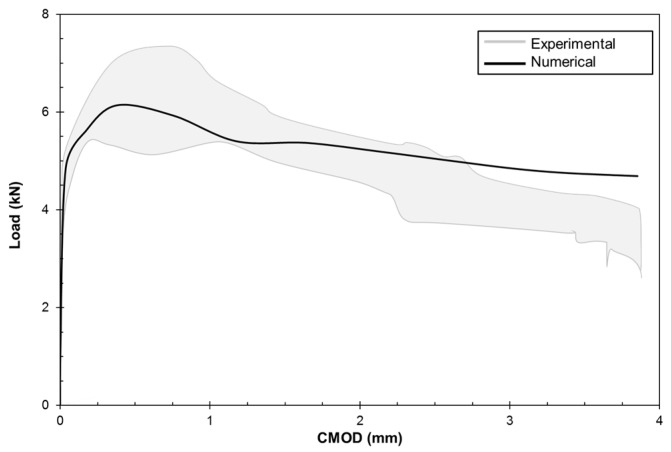
3PBT load-CMOD validation, *V_f_* = 0.6% and *θ* = 30°.

Figure 16 presents the load-CMOD response comparison between the experimental and numerical results. The numerical results are in agreement with the experimental results, especially in the peak and post-peak range, showing the ability of the mesoscale fiber-reinforced lattice–particle model for providing reliable input parameters for macroscale models in a hierarchical homogenization multiscale approach.

## 4. Conclusions

In this work, a lattice–particle model for the analysis of fracture properties of steel fiber-reinforced concrete has been presented. The model has been validated with respect to existing analytical models [43] and experimental data [44], in the uniaxial tensile behavior. Moreover, an experimental campaign was carried out in order to validate the compressive and flexural behaviors.

The characterization of the fiber–matrix interface is of great importance in the presented model. For this reason, pullout tests were carried out and compared to existing experimental results [41,42], and the ability of the model to account for different bonding behaviors has been shown. In this sense, a range for the bonding material properties has been presented.

The fiber-reinforced lattice–particle model is able to account for different volume fractions (*V_f_*) and fiber orientations, characterized by the fiber misorientation angle (*θ*), and the results showed that an increase in *V_f_* leads to an increase in the ductility, while an increase in *θ* has the opposite effect. The model was used to analyze the effect of such parameters on the fracture properties and the results agreed well with the analytical and experimental results, especially for the tensile and compressive strengths. The effect on the ductility was also analyzed by means of the fracture energy and, although the trend is similar to the analytical model by Karihaloo and Lange-Kornbak [43], the predicted values were lower.

An upscaling hierarchical homogenization-based scheme has been presented in order to provide material information at the macroscale by performing virtual tests on the mesoscale model. In this regard, a representative volume element analysis was carried out for FRC in order to determine its size. Moreover, an RVE can be found for the hardening and softening regimes, at least until localization takes place. Thus, conventional multiscale approaches may be followed without loss of generality if these conditions are satisfied. The ability of the lattice–particle model to provide material properties within a multiscale framework has been thus demonstrated by means of 3PBT.

Finally, not only can the presented model be used to provide material properties at the mesolevel, but it can also be connected to other physical models which provide material properties at lower (or larger scales) or information of the material structure, e.g., casting process modeling, opening the door to an Integrated Computational Materials Engineering (ICME) approach [6] for the design of fiber-reinforced cement-based composites.

## Figures and Tables

**Table 1:** Summary of the input parameters required by the fiber-reinforced lattice–particle model.

Phase	Input Properties
Matrix	Elastic: *E_m_*, *E_a_*
	Fracture: *f_t_*, *f_c_*, *c*, *ϕ*, *G_F_*
	Mix information: *w*/*c*, *a*/*c*, *d_max_*
Fiber	Elastic: *E_f_*
	Fracture: *σ*_1_, *σ*_2_, *ε_f_*/*ε_i_*
Bond	Elastic: *E_b_*
	Fracture: *τ*_1_, *τ*_2_, *ε_f_*/*ε_i_*

**Table 2:** Fiber–matrix interface material properties range.

Property	Values
*E_b_* (GPa)	1–10
*τ*_1_ (MPa)	1–10
*τ*_2_ (MPa)	<2
ε*_f_*/ε*_i_*	5–15
